# An Orphan Pheromone Receptor Affects the Mating Behavior of *Helicoverpa armigera*

**DOI:** 10.3389/fphys.2020.00413

**Published:** 2020-04-30

**Authors:** Song Cao, Tianyu Huang, Jie Shen, Yang Liu, Guirong Wang

**Affiliations:** ^1^State Key Laboratory for Biology of Plant Diseases and Insect Pests, Institute of Plant Protection, Chinese Academy of Agricultural Sciences, Beijing, China; ^2^Department of Entomology and MOA Key Lab of Pest Monitoring and Green Management, China Agricultural University, Beijing, China; ^3^Shenzhen Branch, Guangdong Laboratory for Lingnan Modern Agriculture, Genome Analysis Laboratory of the Ministry of Agriculture, Agricultural Genomics Institute at Shenzhen, Chinese Academy of Agricultural Sciences, Shenzhen, China

**Keywords:** *Helicoverpa armigera*, pheromone receptor, CRISPR/Cas9, mating behavior, EAG

## Abstract

The Lepidoptera is the second largest insect order, which has the most extensive knowledge of sex pheromones and mechanisms of pheromone communication since the identification of the first insect pheromone in *Bombyx mori*. In the past 15 years, pheromone receptors have been identified and functionally characterized in many moth species. HarmOR14 is a typical pheromone receptor of *Helicoverpa armigera* which showed no response to the tested pheromones in *Xenopus* oocyte expression system, but its orthologous gene in *Heliothis virescens*, HvirOR14 could be activated by pheromones in the same expression system. To assess the possible functions of OR14 *in vivo*, in this study, we knocked out this gene using CRISPR/Cas9 system and compared the mating behaviors and EAG response to pheromones between the wild type and mutant strains. Our results showed that OR14 mutants did not affect the mating rate or the EAG response to pheromones but could prolong the mating duration and change the mating time in undefined manners, which extends our understanding to this kind of pheromone receptors.

## Introduction

Sex pheromone plays a pivotal role in intraspecific communication between individuals of opposite sex, which likely facilitate the successful survival and reproduction of many species. In most moth species, males heavily rely on species-specific sex pheromones emitted by conspecific females to recognize and orient toward an appropriate mating partner among a large number of sympatric moth species (Linn et al., 1987; Löfstedt et al., 1991; Löfstedt, 1993). The first insect pheromone was identified in the silkmoth *Bombyx mori*, comprises a single component, bombykol (Butenandt et al., 1959; Karlson and Butenandt, 1959), and since then, the pheromone blends from thousands of moths have been uncovered.

The precise reception of pheromone signals is accomplished by male-specific olfactory receptor neurons (ORNs) located in the hair-like cuticular organs-sensilla that are non-randomly distributed on moth antennae (Kanaujia and Kaissling, 1985). So far, extensive progress has elucidated that several molecular elements are involved in the recognition of pheromone at the molecular level in moth including pheromone binding proteins (PBPs) (Zhu et al., 2016), sensory neuron membrane proteins (SNMPs) (Pregitzer et al., 2014), odorant degrading enzymes (ODEs) (Ishida and Leal, 2005; Durand et al., 2011), pheromone receptors (PRs) (Sakurai et al., 2015; Chang et al., 2017) and a highly conserved and broadly expressed (Larsson et al., 2004) odorant receptor coreceptor (Orco) (Nakagawa et al., 2005; Sato et al., 2008). PRs expressed on the dendrites of ORNs determine their selectivity and specificity, suggesting that PR genes are key elements for determining pheromone preference in male moths.

Since the PR genes in moth were initially discovered in *Heliothis virescens* (Krieger et al., 2004) and *B. mori* (Sakurai et al., 2004; Krieger et al., 2005), a great number of PR genes have been identified by homology gene cloning (Forstner et al., 2009; Zhang et al., 2010; Liu C. et al., 2013), whole-genome sequencing (The International Silkworm Genome Consortium, 2008; You et al., 2013; Kanost et al., 2016; Cheng et al., 2017) and high-throughput of transcriptome sequencing (Grosse-Wilde et al., 2011; Bengtsson et al., 2012; Jacquin-Joly et al., 2012; Liu et al., 2012; Steinwender et al., 2015, 2016; Gonzalez et al., 2017; Yuvaraj et al., 2018b; Walker et al., 2019). PRs are a specialized subfamily of odorant receptors (ORs), which showed high degree of conservation between moth PRs reflected in the characteristic clustering of moth PRs in “PR clade” in phylogenetic trees of moth ORs (Engsontia et al., 2014; Steinwender et al., 2015). Moreover, PR genes are thought to be male-biased in most cases (Krieger et al., 2004, 2005) and function to optimally detect pheromones emitted by females. The well functional characterization of PRs leads us to better understand how moths discriminate pheromone cues among the huge number of chemical signals. And it would be more efficient to design attractants or repellents to control moth pests. During the past 15 years, PRs in moths have been widely deorphanized using different heterologous systems as they are efficient and easily available, including the *Xenopus* oocytes expression system (Sakurai et al., 2004; Nakagawa et al., 2005; Wang et al., 2011; Wicher et al., 2017; Hou et al., 2020), the human embryonic kidney (HEK293) cells (Steinwender et al., 2015; Wicher et al., 2017; Yuvaraj et al., 2017, 2018a), the insect cultured cell lines, Sf9 cells (Corcoran, 2011; Liu et al., 2014; Xu et al., 2014), the *Drosophila* empty neuron system (Montagné et al., 2012; de Fouchier et al., 2015; Wang et al., 2016) and so on. However, these heterologous methods are thought to be less accurate because they may not reflect their real scenarios *in vivo*. Recently, it has been reported that PR function has been successfully studied by novel *in vivo* genetic engineering tools, the TALENs system (Sakurai et al., 2015) and the CRISPR/Cas9 system (Chang et al., 2017), which thought to be more efficient and accurate ways for OR deorphanization. With more and more PRs have been deorphanized, the receptors responsible for the recognition of pheromone blends have been revealed in many species. However, there are always some PRs that cannot be activated by any pheromones or analogs in *in vitro* electrophysiological experiments, such as BmorOR4-6 in *B. mori* (Nakagawa et al., 2005), OscaOR6-8 in *Ostrinia scapulalis* (Miura et al., 2010), and CsupOR3&5 in *Chilo suppressalis* (Chang et al., 2015), and so on. Although no ligands have been identified for these PRs, it is inaccurate to conclude that they are non-functional PRs for moths. OR11 in *H. virescens* and *Helicoverpa armigera* both did not respond to sex pheromones, but its orthologous gene in *Operophtera brumata* could be activated by pheromones (Zhang et al., 2016), which meant OR11 in *H. virescens* and *H. armigera* may also have some unexpected functions. Cattaneo found that CpomOR1 from *Cydia pomonella* showed no response to any tested pheromones or plant volatiles when expressed in HEK293 cells (Cattaneo et al., 2017), however, when Garczynski knocked out this gene and found that the fecundity and fertility of female moth were both affected (Garczynsk et al., 2017). These examples lead us to question the roles of these PRs to moth behaviors.

*H. armigera* is one of the greatest economic pest species of modern agriculture in the Old world, which is throughout temperate and tropical regions of Asia, Africa, Australia, Europe, and Oceania (Armes et al., 1992). The sex pheromones of the species are a blend of several components, comprise a major compound, Z11-16:Ald and several minor compounds, Z9-16:Ald, Z9-14:Ald, et al. (Kehat and Dunkelblum, 1990; Zhang et al., 2012). It has been reported that a mixture of Z11-16:Ald and Z9-16:Ald with a ratio from 99:1 to 90:10 caused a significant increase in trap catch of male *H. armigera* (Nesbitt et al., 1979; Kehat et al., 1980; Kehat and Dunkelblum, 1990). And Z9-14:Ald was shown opposite behavioral effects in different concentrations when combined with other pheromone components (Kehat and Dunkelblum, 1990; Zhang et al., 2012; Wu et al., 2015). In previous study, we have identified seven PR genes (*OR6*, *OR11*, *OR13*, *OR14*, *OR14b*, *OR15*, and *OR16*) by analyzing antennal transcriptome data and studied their functions by using the *Xenopus* oocytes expression system (Liu et al., 2012; Liu Y. et al., 2013; Yang et al., 2017). We found that OR13 was the receptor for the major pheromone component, Z11-16:Ald, OR6 and OR14b both responded to two minor pheromone compounds, Z9-14:Ald and Z9-16:Ald, and OR16 was narrowly tuned to Z9-14:Ald and a behavioral antagonist, Z11-16:OH, while the other three PRs, OR11, OR14 and OR15 all showed no response to all the pheromones or analogs (Liu Y. et al., 2013; Yang et al., 2017). Similarly, the OR14 in *Helicoverpa assulta* also showed no response to pheromones (Chang et al., 2016). But the OR14 in *H. virescens*, sharing 84.09% sequence identity with HarmOR14, specifically responded to Z11-16:Ac (Wang et al., 2011), a behavioral antagonist to male *H. virescens* (Vickers and Baker, 1997), which was emitted by a closely related species *Heliothis subflexa* (Teal et al., 1981). Therefore, OR14 in *H. armigera* maybe also responsible for recognition of some behavioral relevant chemicals and influence the mating behaviors.

Considering the possible differences of PR function identified from the *in vitro* expression systems and the *in vivo* gene knockout methods, it makes us eager to confirm its function *in vivo*. In this study, we knocked out *HarmOR14* by using the CRISPR/Cas9 technique and compared the mating behavior, including the mating rate, mating duration, mating time, and EAG response to pheromones between the wild type and mutated moths. The results showed that the *HarmOR14* mutants did not significantly affect the mating rate or the EAG responses to pheromones but prolong the mating duration and change the peak mating time compared to the wild type population. Our findings indicate that HarmOR14, although no ligands identified *in vitro*, could partly affect the mating behavior of *H. armigera* in undefined manners, which extends our understanding to this kind of PRs, and may also provide a new candidate target for pest control.

## Materials and Methods

### Insect Culture

*H. armigera* used in all experiments were reared at the Institute of Plant Protection, Chinese Academy of Agricultural Sciences, Beijing, China. Larvae of all strains were fed on an artificial diet at 26 ± 1°C, 55% ± 5% relative humidity with a photoperiod of 14:10 h (light for 06:00–20:00 and dark for 20:00–06:00). Pupae were sexed and individually kept in glass tubes before eclosion. For adults, 10% sucrose solution was supplied daily for diet supplement.

### Genetic Mapping of Pheromone Receptors in *H. armigera* Genome

Pheromone receptors of *H. armigera* containing *OR6*, *OR11*, *OR13*, *OR14*, *OR14b*, *OR15*, and *OR16* are available in NCBI databases under the accession number MN399770 to MN399776, respectively. Based on the genome sequence data of *H. armigera* (Pearce et al., 2017), all the PR sequences were analyzed by Local BLAST in Bioedit software to locate them in *H. armigea* genome. The loci information of PR genes were used to generate a visible map using the tool GSDS2.0^1^.

### Sequence Analysis of *HarmOR14* and Preparation of Single Guide RNA (sgRNA)

Sequence alignment of *OR14* from *H. armigera*, *H. assulta*, and *H. virescens* were conducted using DNAMAN 8 software (Version 8, Lynnon Biosoft, Quebec, Canada), and the transmembrane domains were predicted on TOPCONS website^2^.

To obtain a proper target for gene knockout, 12 adult moths were used to extract genomic DNA individually. The genomic DNA was then used as templates to amplify a DNA fragment with a pair of gene specific primers. The forward primer was in exon 2 and the sequence was: 5′-GATTACCCCTACATGATTTTGTGTC-3′; the reverse primer was in exon 3 and the sequence was: 5′-AGAAGTAAAGAGAATGTTCAAACG-3′. The 25 μL PCR mixture contained 12.5 μL 2 × Taqmix, 0.5 μL F primer, 0.5 μL R primer, 2.5 μL DNA template and 9 μL RNase-free H_2_O. The PCR was conducted under the following conditions: 95°C for 3 min, 38 cycles of 95°C for 30 s, 58°C for 30 s and 72°C for 1 min, followed by a final extension period of 72°C for 5 min. The PCR products were ligated into pEASY-T3 vector and sent to sequence the genotypes. According to the principle 5′-G(19N)NGG-3′, a 23 bp-long conserved sequence 5′-GGGTGCTTCATAACGACTATTGG-3′ located in exon 2 of *HarmOR14* was selected as the sgRNA target site (Figure 2B). The small sequence was used to do Local BLAST in *H. armigera* genome to avoid off-target effects.

Before synthesis of the sgRNA, a DNA template was prepared by PCR according to the manufacturer’s instruction (GeneArt Precision gRNA Synthesis Kit, Thermo Fisher Scientific, Pittsburgh). The PCR was performed with a pair of designed primers (the forward primer: GAAATTAATACGACTCACTATAG + target sequence, the reverse primer: TTCTAGCTCTAAAAC + target sequence reverse complement) and other reagents provided in the kit under the conditions: 98°C for 10 s, 32 cycles of 98°C for 5 s and 55°C for 15 s, followed by a final extension of 72°C for 1 min. The sgRNA was generated with an *in vitro* transcription reaction. After that, the sgRNA was purified by a gRNA Clean Up Kit and diluted into working concentration with nuclease-free water.

### Eggs Microinjection

The egg collection and preparation were conducted as described by Chang et al. (2017). A mixture of sgRNA (200 μg/μL) and Cas9 protein (200 μg/μL, GeneArt^TM^ Platinum^TM^ Cas9 Nuclease, Thermo Fisher Scientific, Shanghai, China) were injected into individual eggs using a FemtoJet and injectMan NI 2 microinjection system (Eppendorf, Hamburg, Germany). The egg collection and microinjection were completed within 2 h. The injected eggs were maintained at 26 ± 1°C, 55% ± 5% RH for 3–4 days until hatching.

### HarmOR14 Mutants Screening

When the hatched larvae were reared to adults (F0), they mated with *H. armigera* of wild type. After oviposition, the eggs were collected. When the F1 grew into adults, the middle leg was removed and used to extract genomic DNA, respectively, using a TIANamp Genomic DNA Kit (TIANGEN, Beijing, China). The genomic DNA was used as templates to amplify a 935 bps-long fragment including the target site with the above-mentioned primers under the same conditions. The amplified fragments were directly sequenced with the forward primer. The PCR products showing multiple peaks in the sequence chromatogram were ligated into a pEASY-T3 vector (TransGen Biotech, Beijing, China) and sent to sequence the detailed genotypes. The screened moths with same genotypes were mixed and allowed to produce homozygous mutants.

### Mating Assays

The 2- to 3-day-old virgin adults were used for the mating assays with a controlled condition of 26 ± 1°C, 55% ± 5% relative humidity. When scotophase was coming, a pair of male and female were put into a plastic container, 10.5 cm high and 8 cm in diameter, and the behavior during the dark period was recorded with a night vision video camera (SONY, Japan). A piece of degreasing cotton containing 10% sucrose solution was put in the bottom of the container to supply nutrition. In experimental group, we used the HarmOR14 mutated males and wild type females, while in control group, we used both wild type males and females. In the mating experiment, the behavior of 15 pairs of moths in the whole scotophase were recorded with one camera and treated as one group (Figure 3A). By observing the video, we observed and counted the mating state of each pair of moths. The mating rate of each group was calculated by dividing the total number of pairs in each group with the number of mated pairs in the group and then we got one mating rate for each group. At the same time, the mating start time and end time of each pair of mated moths in all groups were recorded and the mating duration was calculated. For the mating time, we counted the number of pairs started to mate in each hour from 20:00 to 06:00 in all groups, and calculated the rate of mating pairs in each hour and then drew a line graph with the data by using the GraphPad 8 software. In the behavioral experiments, 22 groups of wild type and 13 groups of OR14 mutated moths were tested.

### Pheromones and Electroantennogram (EAG) Recording

Pheromones used in EAG experiment including Z11-16:Ald, Z9-14:Ald, and Z9-16:Ald were purchased from Nimrod Inc (Changzhou, China) with more than 96% purity. Before use, pheromones were dissolved in paraffin oil and diluted to six different concentrations, 0.01 μg/μL, 0.1 μg/μL, 1 μg/μL, 10 μg/μL, 100 μg/μL, and 200 μg/μL, and stored at −20°C until use.

The EAG experiment was conducted as previously described (Cao et al., 2016). The cut antenna of 2- to 3-day-old virgin males was inserted between two glass electrodes filled with 0.1 M KCl solution. The reference electrode and the recording electrode were connected to the two sides of the antenna, respectively. A piece of filter paper (0.5 cm × 5 cm) loaded with 10 μL each solution was inserted into a Pasteur pipette to deliver the stimuli. 10 μL of paraffin oil was used as blank control. Each antenna was stimulated with paraffin oil and a single pheromone of the different concentrations in an increasing order. Each pheromone was repeated for at least 20 times. The continuous air flow (30 mL/s) and the odor stimulating flow (0.2 s at 10 mL/s) were produced and controlled by a stimulus controller (CS-55, Syntech, Netherlands). The stimulated signals were amplified with a 10 × AC/DC headstage preamplifier (Syntech, Netherlands) and acquired with an Intelligent Data Acquisition Controller (IDAC-4-USB; Syntech, Netherlands). The signals were recorded with a Syntech EAG-software.

### Statistics

The mating rate, mating duration and relative EAG responses to a certain concentration of each pheromone between the wild type and mutant were analyzed by the Student’s *t*-test. All statistical analyses were performed using SAS V8 for window software. Statistical significance was determined at α = 0.05 level.

## Results

### Genetic Mapping of Pheromone Receptors in *H. armigera* Genome and Sequence Analysis of *HarmOR14*

**FIGURE 1:**
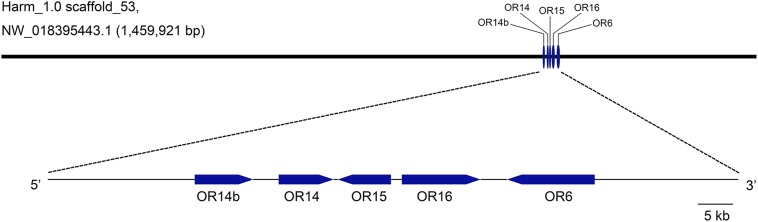
Genetic mapping of pheromone receptors in *H. armigera* genome. Five PR genes including *OR14b*, *OR14*, *OR15*, *OR16*, and *OR6* are located in a same scaffold (Harm_1.0 scaffold_53, NW_018395443.1) in tandem arrays. The visible map was generated using the tool GSDS2.0 (http://gsds.cbi.pku.edu.cn) according to the loci information of PR genes.

To better understand the evolutionary relationships of PRs in *H. armigera*, the loci information was analyzed and used to map these PRs in Genome sequences (GenBank assembly accession: GCA_002156985.1). It showed that *OR13* was not found in the genome and *OR11* was in scaffold_9, while the other five PRs including *OR6*, *OR14*, *OR14b*, *OR15*, and *OR16* were all mapped in scaffold_53 in tandem arrays (Figure 1). Besides, OR14 was located between OR14b and OR15.

**FIGURE 2:**
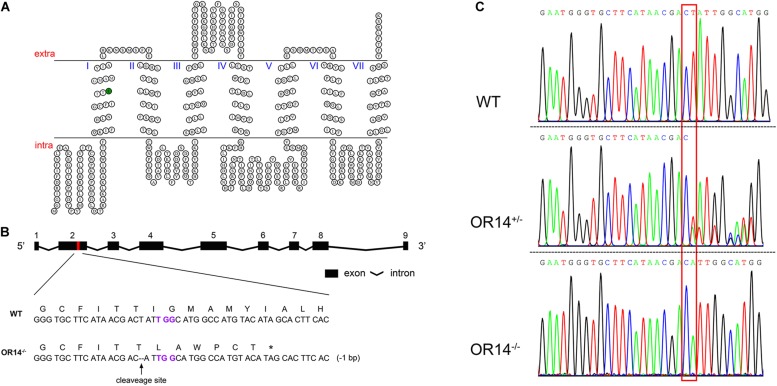
CRISPR/Cas9-based knock out of *H. armigera OR14*. **(A)** The HarmOR14 protein possesses seven transmembrane domains. The seven transmembrane domains were predicted on TOPCONS website (http://topcons.net/). The nucleotide deletion was generated in the first transmembrane domain (green circle). **(B)** The *HarmOR14* gene contains nine exons in genome and the exon 2 was targeted for CRISPR single-guide RNA (sgRNA) design (red region). PAM site was indicated in purple and the deletion was indicated as dash lines. The mutation caused a frameshift at codon 75 and a premature stop codon, giving rise to a truncated protein of 80 amino acids. **(C)** The three sequencing chromatograms indicated three genotypes, WT (top), heterozygote (middle) and homozygote (bottom), respectively, and the mutation site was shown with a red frame.

**FIGURE 3:**
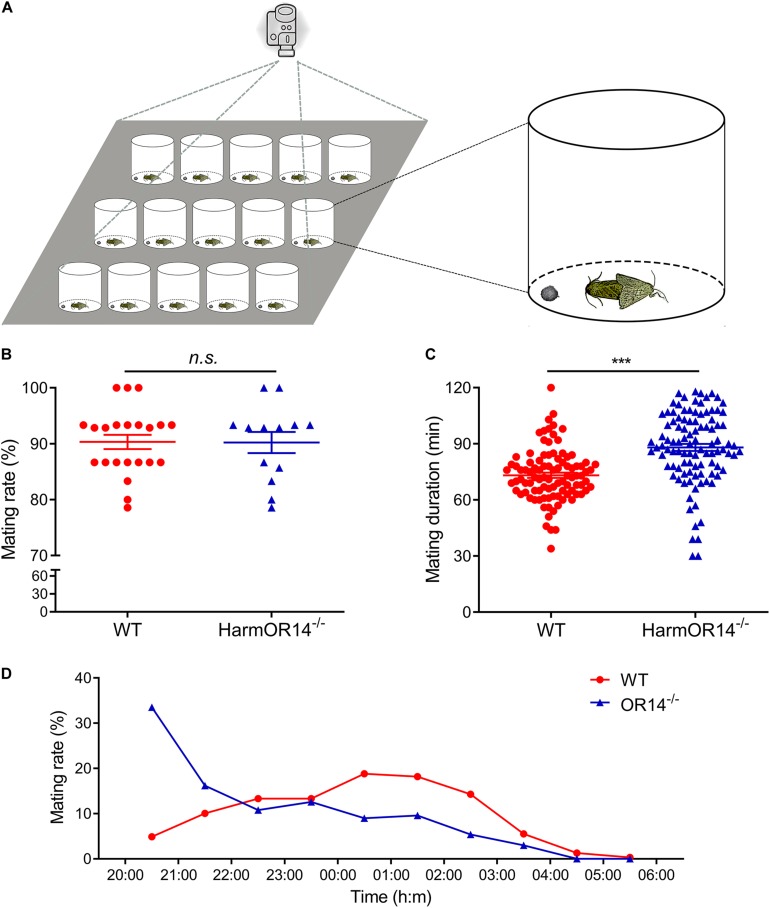
The mating behavior of wild type and OR14 mutant strains. **(A)** Schematic representation of behavioral experiment design. **(B)** Scatter-dot plot comparing the mating rate of wild type (left, red) and OR14 mutant (right, green) strains. Each dot represents the mating rate of one group. Error bars indicate SEM. *n* = 22 and 13, respectively. Student’s *t*-test, df = 33, *t* = –0.05, *P* = 0.962. **(C)** Scatter-dot plot comparing the mating duration of wild type (left, red) and OR14 mutant (right, green) strains. Each dot represents the mating duration of one pair of successful mating moths. *n* = 96 and 102, respectively. Student’s *t*-test, df = 184, *t* = 6.23, *P* < 0.001. *n.s.*, no significant difference; *P < 0.05; **P < 0.01; ***P < 0.001. **(D)** Line graph showing the mating rate in each hour from 20:00 to 06:00 of wild type (red) and OR14 mutant (green) strains.

The full length of *OR14* is 1323 bps long and encode 440 amino acid residues. As reported in other insect ORs, HarmOR14 also possess seven transmembrane domains (Supplementary Figure S1). Sequence alignment of HarmOR14 with its two orthologous HassOR14 and HvirOR14 indicates that the three ORs share 93.56% of amino acid identity (Supplementary Figure S1).

### CRISPR-Cas9 Generation of *OR14* Mutant Strain

To study the function of HarmOR14 *in vivo*, we applied CRISPR-Cas9 to knockout this gene and obtain a premature and non-functional protein. According to the genomic arrangement of HarmOR14 cluster (Figure 2B), a 20-bp long sequence followed by PAM sequence located in the first transmembrane domain was selected to target the gene (Figure 2A). The specific sgRNA and Cas9 protein were co-injected into newly produced eggs of *H. armigera*. Among 500 approximately injected eggs, 186 hatched larvae were reared to adult and mass crossed with wild type moths to produce offspring (F1). Genomic DNA of each F1 adults was extracted with one of middle legs and used as templates to amplify a 935 bps-long fragment containing the target site and then sent to sequence the genotypes. To create a homozygous *HarmOR14* mutated strain, in this study, the heterozygote adults with a 1-bp deletion at exon 2 (Figures 2B,C) were selected and mass crossed to produce F2. About 100 adults of F2 were genotyped and only the individuals with homozygous mutations were pooled to produce homozygous mutated strain. The mutation caused a frameshift at codon 75 located at the N-terminal intracellular segment (Figure 2A), and a premature stop codon, giving rise to a truncated protein of 80 amino acids (Figures 2A,C).

### Comparation of Mating Behavior of Wild Type and *OR14* Mutated Moth

To investigate if OR14 has any effects on the mating behavior of *H. armigera*, we compared the mating rate, mating duration and mating time of OR14 mutant with wild type moths. Considering OR14 is only expressed in male antenna, we speculated that OR14 only affected mating behaviors of males. Therefore, in the experimental group, we used the OR14 mutated males, and used wild type males in control group, and the females used in the behavioral experiments were wild type. One male and one female were put into a plastic container around 20:00, and the mating behaviors were recorded with a camera until 6:00 in the next morning (Figure 3A). Fifteen containers were recorded with one camera and treated as one group.

We did the behavioral experiments with 22 groups of wild type moths and 13 groups of OR14 mutants. The results showed that the mating rate were 90.32% and 90.22% for the wild type moths and OR14 mutants, respectively, which showed no significant difference between the two groups (Figure 3B; df = 33, *t* = −0.05, *P* = 0.962).

Then we analyzed the mating duration of each successful mating pairs and the results indicated that the average mating duration of OR14 mutated moths was 87.89 min which was much longer than 73.20 min of the wild type moths (Figure 3C; df = 184, *t* = 6.23, *P* < 0.001), by prolonging 20.1% of mating duration. For the mating time, we found that in the wild type population, the peak mating time was 00:00 to 02:00 (∼37%), but for OR14 mutants, it occurred in the first 2 h, 20:00 to 22:00 (∼49.7%) (Figure 3D), especially in the first hour (∼33.5%), which means the peak mating time ahead in the OR14 mutants.

### Comparation of EAG Response to Pheromones of Wild Type and *OR14* Mutant Strain

**FIGURE 4:**
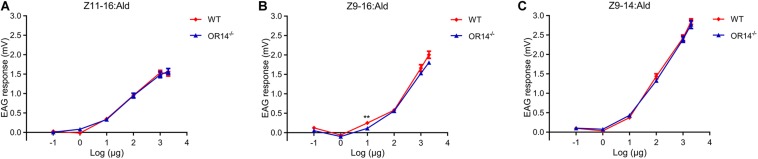
EAG responses of wild type and OR14 knockout males to pheromone blends in different concentrations. **(A–C)** Dose-response curves of wild type (green) and OR14 mutated male (red) antennae stimulated with Z11-16:Ald, Z9-16:Ald and Z9-14:Ald, respectively. Error bars indicate SEM. *n* = 18–20. *P < 0.05; **P < 0.01; ***P < 0.001.

Considering OR14 did not affect the mating rate but the mating duration and the mating time of *H. armigera*, we speculated that OR14 may affect the sensing of pheromones that regulate the beginning or termination of mating behavior. Therefore, we tried to compared the EAG response to different concentrations of pheromones between the wild type strain and OR14 mutant strain. The results indicated that as the increasing of pheromone concentrations from 1 μg, the EAG responses gradually increased (Figures 4A–C), but there was no significant difference of EAG responses between the two strains to certain concentration of pheromones except for 10 μg of Z9-16:Ald (Figures 4A–C).

## Discussion

In moth, the intraspecific communication is mediated by sex pheromone. The PR is a vital element in pheromone communication system as it directly binds to pheromone molecules and leads to signals transduction. Functional characterization of PRs in various moth species will help to unravel the evolution of species-specific pheromones and corresponding receptors, as well as contribute to developing efficient methods for pest control. To deorphanize the function of PRs, several heterologous expression systems have been developed and employed, typically for their convenience and efficiency. However, the assessment of ligand specificities of PRs with *in vitro* methods is under suspicion because the functions of PRs can be affected by the set of compounds tested and some other cellular proteins *in vivo*, moreover, the exist inherent differences between different heterologous systems may also affect the receptor’s ability to respond to ligands. For example, *EposOR1* of *Epiphyas postvittana* is clustered into PR clade in the phylogenetic tree (Jordan et al., 2009), when expressed in Sf9 cells, it can be activated by several plant volatiles but not pheromones (Jordan et al., 2009), however, when expressed in HEK293 cells, it was only activated by pheromone compounds but not the plant volatiles functioning in Sf9 cells (Corcoran, 2011). Yuvaraj et al. (2017) and Hou et al. (2020) compared the PR function of *Eriocrania semipurpurella* using two different heterologous systems, HEK293 cells and *Xenopus* oocytes, and it turned out that the PR responses exhibited some differences between the two different systems. Therefore, the *in vivo* genome editing methods, such as TALENs (Sakurai et al., 2015) and CRISPR/Cas9 systems (Chang et al., 2017; Garczynsk et al., 2017) are introduced to confirm the PRs’ function.

Seven PR genes of *H. armigera* have been identified previously (Liu et al., 2012; Zhang et al., 2015) and five of them including *OR6*, *OR14*, *OR14b*, *OR15*, and *OR16* are located in a single scaffold (Scafford_53) in tandem arrays (Figure 1). Besides, these PR genes are more closely related to each other than any other ORs in a phylogenetic tree (Zhang et al., 2015), more likely gene duplication. This phenomenon is similar with that in other two heliothine species, *H. virescens* and *H. subflexa*, where four orthologous PRs of *H. armigera*, including *OR6*, *OR14*, *OR15*, and *OR16*, are tight linkage in a single quantitative trait locus (QTL) (Gould et al., 2010). It means that as the evolution of heliothine moths, these four PRs are still maintained their locus features although a new PR gene, OR14b was duplicated in *Helicoverpa* species. Functional study results showed that HarmOR14 could not be activated by pheromones in the *Xenopus* oocyte system (Liu Y. et al., 2013), which is different from its orthologous PR, HvirOR14 (Wang et al., 2011). Considering the possible shortcomings of the *in vitro* expression system, in this study, we knocked out this gene using the CRISPR/Cas9 system, and compared the mating behaviors and EAG responses to pheromones between the mutated and wild type moths. It turned out that the OR14 could not affect the mating rate or the EAG responses to pheromones but modulated the mating duration and the peak mating time of *H. armigera* (Figures 3, 4).

It is known that the sexual behavior of moths can be regulated by changes of pheromone ratios (Baker, 2008) or proteins in the pheromone sensing system (Sakurai et al., 2015; Chang et al., 2017). In this study, the pheromone components released by females are the same in experimental group and control group because we used the wild type females in both groups in the behavioral experiments. So, the changes of mating behavior possibly happened due to the absence of *HarmOR14*. In this study, we proved that the OR14 mutants did not affect EAG response to three most important pheromone compounds (Figure 4), but it still has the possibilities to be activated by other chemicals. It has been reported that some lepidoptera moths use both type I and type II pheromones (Cabrera et al., 2001; Millar et al., 2005; Gibb et al., 2007), and PR can also be activated by both type of pheromones (Zhang and Löfstedt, 2013; Zhang et al., 2016). Therefore, OR14 may affect the recognition of this kind of pheromones. Besides, studies reported that neurons grouped in a same sensilla could inhibit each other and modulate olfactory behavior (Su et al., 2012; Zhang et al., 2019), so we speculated that the changes in mating behavior possibly regulated by direct or indirect effects of OR14. But all these are guesswork, and we will try to clarify the exact mechanism in our future studies.

Together, our findings provide evidence that a PR in moth without ligands been identified can modulate the mating behavior to some extent, which broaden our understanding to this kind of PRs, and may provide a new candidate target for pest control.

## Data Availability Statement

All datasets generated for this study are included in the article/Supplementary Material.

## Author Contributions

GW, YL, and SC designed the experiments, SC and TH performed the experiments. SC wrote the manuscript and analyzed the data. GW, YL, and JS revised the manuscript.

## Conflict of Interest

The authors declare that the research was conducted in the absence of any commercial or financial relationships that could be construed as a potential conflict of interest.

## Footnotes

1 http://gsds.cbi.pku.edu.cn

2 http://topcons.net/

## Supplementary Material

FIGURE S1 Sequence alignment of *OR14* in *H. armigera*, *H. assulta*, and *H. virescens*.
