# Supplementary figures and images for: An Orphan Pheromone Receptor Affects the Mating Behavior of Helicoverpa armigera
Source: Front Physiol. 2020 Apr 30;11:413. doi: 10.3389/fphys.2020.00413 (PMC7204811; doi:10.3389/fphys.2020.00413)

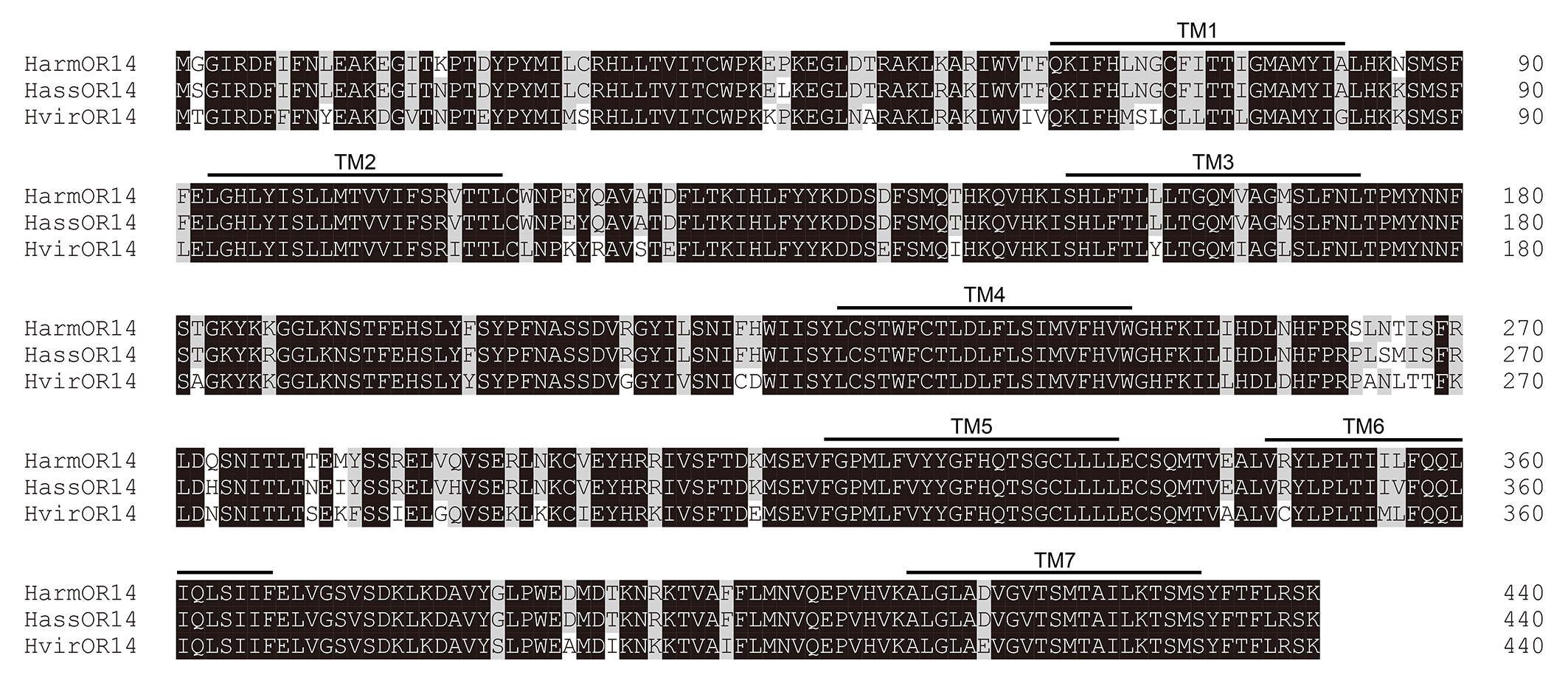

Supplement: FIGURE S1 — Sequence alignment of OR14 in H. armigera, H. assulta, and H. virescens. [file Image_1.TIF]
